# Experimental infections of mosquitoes with severe fever with thrombocytopenia syndrome virus

**DOI:** 10.1186/s40249-017-0282-6

**Published:** 2017-06-01

**Authors:** Shu-Yi Liang, Hong-Liang Chu, Xi-Ling Guo, Wei Wang, Hong-Na Chen, Yu-Fu Zhang, Yin Chen, Tao Wu, Chang-Jun Bao, Ming-Hao Zhou

**Affiliations:** 1 Jiangsu Provincial Center for Disease Control and Prevention, No. 172 Jiangsu Road, Nanjing, People’s Republic of China; 2 Jiangsu Institute of Parasitic Diseases, No. 117 Yangxiang, Meiyuan, Wuxi, People’s Republic of China

**Keywords:** Severe fever with thrombocytopenia syndrome virus (SFTSV), Vector, *Culex pipiens pallens*, *Aedes aegyptis*, *Anopheles sinensis*, Real-time RT-PCR

## Abstract

**Background:**

Severe fever with thrombocytopenia syndrome (SFTS) is a newly identified emerging infectious disease, which is caused by a novel bunyavirus (termed SFTSV) in Asia. Although mosquitoes have not been identified as the primary vectors, as revealed by epidemiological surveys, their role in transmitting this SFTSV as a suspicious vector has not been validated.

**Findings:**

In this study, we conducted experimental infections of mosquitoes with SFTSV to examine the role of mosquitoes in the transmission of the virus. We did not detect viral replication in *Culex pipiens pallens*, *Aedes aegyptis* and *Anopheles sinensis* as revealed by qRT-PCR assay. In addition, we failed to isolate SFTSV from the Vero cells cultured with suspensions of SFTSV-infected mosquitoes.

**Conclusion:**

The results of the present study demonstrate little possibility that mosquitoes act as vectors for the emerging pathogen SFTSV.

**Electronic supplementary material:**

The online version of this article (doi:10.1186/s40249-017-0282-6) contains supplementary material, which is available to authorized users.

## Multilingual abstracts

Please see Additional file 1 for translations of the abstract into six official working languages of the United Nations.

## Background

Since 2009, sporadic cases with clinical manifestations of acute onset of fever, low white blood cell and platelet counts, high levels of alanine and aspartate transaminases, and proteinuria, have been found in China [1]. A novel bunyavirus, which was then termed severe fever with thrombocytopenia syndrome virus (SFTSV) in 2011, was found to be associated with this disorder [2, 3]. Moreover, confirmed SFTS cases have been reported from Japan and South Korea in recent years [4, 5]. Severe fever with thrombocytopenia syndrome (SFTS), an emerging infectious disease with mortality up to more than 10%, has become an increasingly important public health concern in China [6].

As a novel virus identified in the genus *Phlebovirus* of the family Bunyaviridae, little is known about the natural transmission cycle. *Haemaphysalis longicornis*, a widely distributed tick species, has been supposed to act as a vector of SFTSV [7]. However, most patients denied history of tick bite before their onset of SFTS [8]. In addition, epidemiological analyses of a cluster of cases infected with SFTSV showed evidence of person-to-person transmission of the virus through direct blood contact with the patients [9–12]. Nevertheless, there is little knowledge on the vector during the person-to-person transmission cycle of SFTSV. Mosquitoes are probably the most common vectors of infectious diseases [13], and they are identified as major vectors for transmission of multiple Bunyaviridae viruses [14]. The present study aimed to investigate the role of mosquitoes in the transmission of SFTSV.

## Methods

This study was approved by the Ethical Review Committee of Jiangsu Provincial Center for Disease Prevention and Control (JSCDC). All animal experiments were performed in strict accordance with the Guidelines for Laboratory Animal Care and Management of China [15]. The SFTSV strain JS-2010-014 used in this study was initially isolated from a patient in 2010 in a bio-safety level-2 (BSL-2) facility of Jiangsu Province.

Experimental infection was performed in a BSL-2 laboratory in JSCDC. After starvation for 12 h, three dominant mosquito species *Culex pipiens pallens*, *Aedes aegypti* and *Anopheles sinensis* were fed with sugar water, SFTSV strain JS-2010-014, and blood of guinea pigs (Nanjing Anlimo Technology Co., Ltd.; Nanjing, China) at a ratio of 1∶1∶6 (blood mixture sample) for 4 h. Then, each species was randomized to 5 cages, of 100 to 150 mosquitoes in each cage, and was fed with sugar water for 4, 24, 48, 72, and 96 h, respectively, while uninfected mosquitoes served as controls. At assigned time points, 20 female mosquitoes of each species were collected, washed three times with physiological saline and ground with maintenance medium to yield mosquito suspensions.

SFTSV RNA was isolated from mosquito suspensions, and viral loads were determined by using TaqMan quantitative real-time PCR (qRT-PCR) assay as described previously [16]. PCR cycle threshold (Ct) values of 35 or less were considered positive, while Ct value of > 35 was defined negative. All samples were subjected to SFTSV RNA detection as described above.

Once qRT-PCR showed positive, the samples were used for virus isolation. SFTSV-positive mosquito suspensions were centrifuged, and the supernatant was seeded onto single-layer African green monkey kidney Vero cells. Cytopathic effect of Vero cells was assessed using indirect immunofluorescence assay (IFA). Vero cells were grown on an 8-well Millicell EZ slide (Millipore; Billerica, MA, USA) at 37 °C for 36 h, and cells were fixed by treatment with acetone for at 20 °C 10 min. Mouse anti-SFTSV NP polyclonal antisera were incubated with the fixed cells at 37 °C for 30 min. Bound antibodies were detected using fluoresce in isothiocyanate (FITC)-conjugated anti-mouse antibodies (KPL; Gaithersburg, MD, USA) that were diluted by PBS containing 0.01% Evens blue, and observed under an Eclipse TS100 inverted fluorescence microscope (Nikon; Kanagawa, Japan). Cell passage was performed blindly for three passages. Loads of SFTSV at each passage were assessed using qRT-PCR assay. Normal Vero cells without inoculation served as a blank control, and SFTSV-infected Vero cells were used as a positive control.

## Findings

**Fig. 1:**
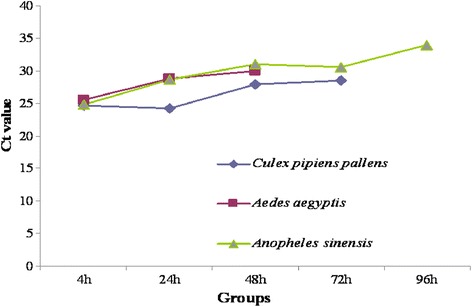
RT-PCR assay detects viral loads of SFTSV in *Cx. Pipiens allens*, *Ae. Egypti* nd *An. inensis* at different breeding time following feeding blood mixture samples

**Fig. 2:**
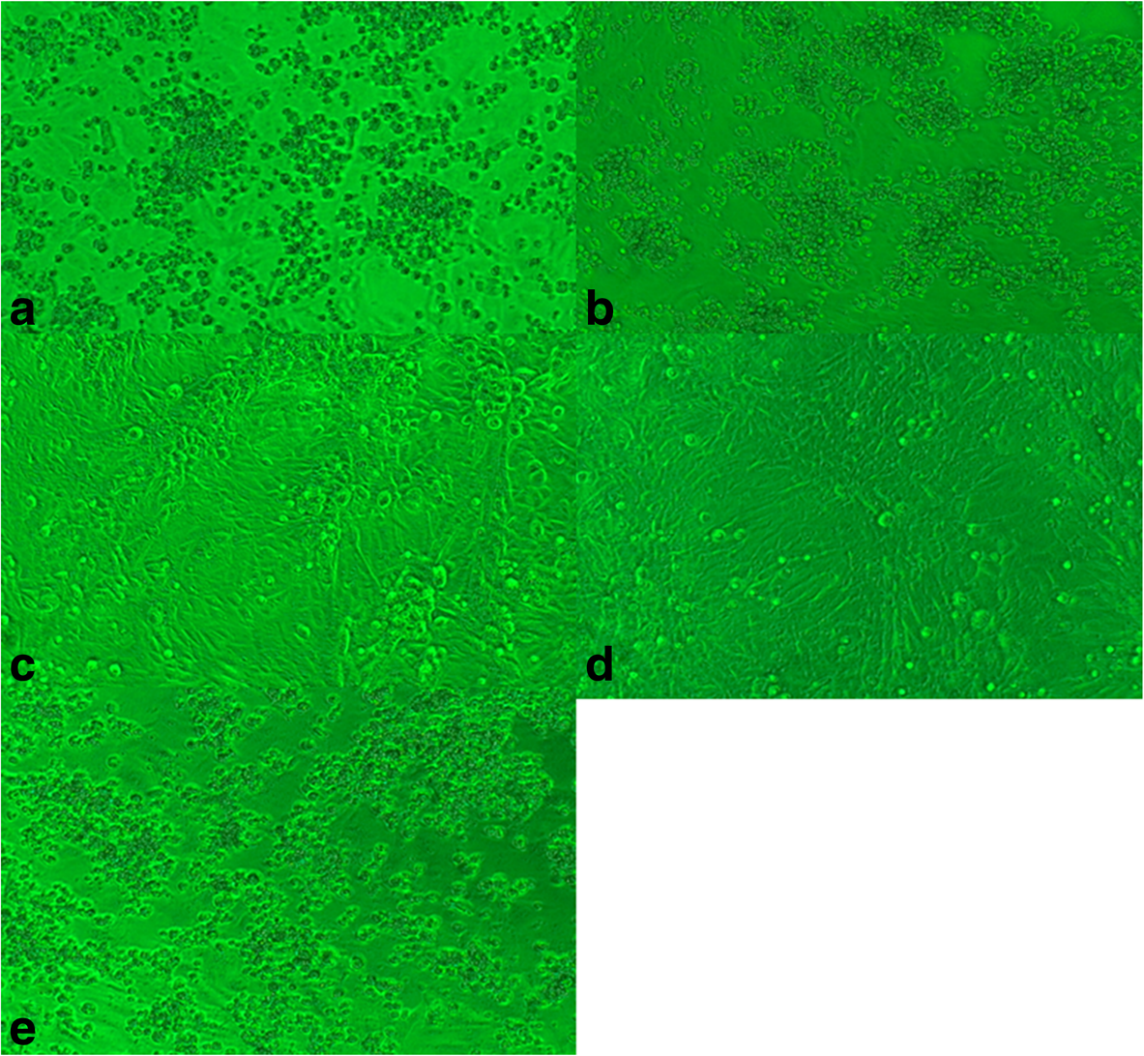
ytopathic effect of Vero cells. **a** Cytopathic effect observed in Vero cells infected with SFTSV JS-2010-014 strain; **b** Cytopathic effect observed in Vero cells inoculated with blood mixture sample; **c** Cytopathic effect seen in normal Vero cells; **d** Cytopathic effect found in Vero cells inoculated with *Cx. Pipiens allens* uspensions following 24 h feeding of blood mixture samples; **e** Cytopathic effect found in Vero cells inoculated with *x. pipiens allens* uspensions following 4 h feeding of blood mixture samples

qRT-PCR assay revealed positive SFTSV in *Cx. pipiens pallens* that was fed with sugar water for 4, 24, 48, and 72 h, *Ae. Aegypti* given sugar water for 4, 24, and 48 h, and *An. Sinensis* fed with sugar water for 4, 24, 48, 72 and 96 h (Table 1). An increasing tendency of Ct values was observed in all three mosquito species with the extension of feeding (Fig. 1), indicating no viral replication detected in mosquitoes. Following inoculation of the supernatant of SFTSV-positive mosquito suspensions, Vero cells developed cytopathic effect in all three passages of *Cx. pipiens pallens* that was fed with blood mixture samples for 4 h, and qRT-PCR detected positive SFTSV. However no cytopathic effect was seen in Vero cells seeded with other two *Ae. Aegypti* or *An. Sinensis* suspension supernatant, and negative SFTSV was detected by qRT-PCR assay (Fig. 2a-e).

**Table 1:** qRT-PCR assay detects viral loads of SFTSV in *Culex pipines pallens*, *Aedes aegyptis* and *Anopheles sinensis* following different breeding time

Breeding time (h)	Ct value		
	*Culex pipiens pallens*	*Aedes aegyptis*	*Anopheles sinensis*
4	Positive (24.59)	Positive (25.58)	Positive (24.84)
24	Positive (24.15)	Positive (28.82)	Positive (28.69)
48	Positive (27.89)	Positive (30.03)	Positive (31.06)
72	Positive (28.49)	Negative (>35)	Positive (30.61)
96	Negative (>35)	Negative (>35)	Positive (33.94)

Identification of a vector requires an evidence of isolation of pathogen and evidence of vector transmission capability and ecology [17]. In this study, artificial infection of mosquitoes with SFTSV was performed to assess the capability to transmit the virus. qRT-PCR assay showed that three mosquito species *Cx. Pipiens pallens*, *Ae. Aegyptis* and *An. sinensis* were successfully infected with artificial blood mixture sample containing SFTSV, and viral loads gradually declined with the extension of breeding time after mosquitoes were fed with SFTSV. In addition, no viral replication was detected However, SFTSV was isolated from Vero cells inoculated with SFTSV-positive *Cx. Pipiens pallens* suspensions, while the virus was not isolated from cells seeded with suspension supernatant of *Ae. Aegyptis* and *An. sinensis*. The results of the current study demonstrate that the viral ability of SFTSV almost disappears in mosquitoes, and no viral replication is detected after 4 to 24 h of virus feeding, which proves that mosquitoes have no ability to transmit SFTSV. Isolation of SFTSV from Vero cells inoculated with SFTSV-positive *Cx. Pipiens pallens* suspensions after 4-h virus feeding may be caused by incomplete wash of SFTSV, which is mechanically carried on the body surface of *Cx. Pipiens pallens*.

## Conclusions

Our findings demonstrate that mosquitoes are not vectors for transmission of SFTSV. Currently, ticks are widely accepted as vectors of SFTSV [1, 2, 18–20]. However, further studies are needed to validate previous hypothesis. Since SFTS caused by SFTSV has a high fatality, and is an increasingly public health concern, health education pertaining to knowledge on correct, effective prevention of vector control should be strengthened. Notably, individual protection targeting high-risk population is of great significance to guide the prevention of SFTS. In addition, health sections require intensification of monitoring and clinical therapy of this emerging infectious disease and the ability of early identification and early diagnosis should be improved to reduce the incidence of SFTS and protect human health.

## Additional file

Additional file 1: Multilingual abstracts in the six official working languages of the United Nations. (PDF 785 kb)

## Abbreviations

PCR: Polymerase chain reaction
SFTS: Severe fever with thrombocytopenia syndrome
SFTSV: Severe fever with thrombocytopenia syndromevirus
