# Supplementary material for: Experimental infections of mosquitoes with severe fever with thrombocytopenia syndrome virus
Source: Infect Dis Poverty. 2017 Jun 1;6:78. doi: 10.1186/s40249-017-0282-6 (PMC5452404; doi:10.1186/s40249-017-0282-6)
Supplement: Additional file 1: — Multilingual abstracts in the six official working languages of the United Nations. (PDF 785 kb) [file 40249_2017_282_MOESM1_ESM.pdf]

Translation of the abstract into the five official working languages of the United Nations

إصابة البعوض بحمى شديدة بواسطة عدوى تجريبية بفيروس متلازمة نقص صفيحات الدم

شو يي يانغ، هونغ ليانغ تشو، شي لينغ غوه، وانغ وى، هونغ نا تشن، يو فو تشانغ، يين تشن، تاو وو، تشانغ جون باو، مينغ هاو تشو

ملخّص:

تقديم عام: تُعدّ الحمّى الشديدة الناتجة عن متلازمة نقص صفيحات الدم (SFTS) كمرض معدٍ ناشئ تمّ تشخيصه حديثا، وهو ناتج عن فيروسات بانيوية نادرة (تُسمّى SFTSV) في آسيا. بالرغم من أنه لم يتم تحديد البعوض كناقل رئيسي، كما تم تداوله في الاستبيانات الوبائية، إلا أنه لم يتم تأكيد دورها المحتمل كناقل بعد.

النتائج: في هذه الدراسة، قمنا إصابة البعوض بحمى شديدة بواسطة عدوى تجريبية بفيروس متلازمة نقص صفيحات الدم للتأكّد من دور البعوض كناقل لهذا الفيروس. واعتمادا على تقنية qRT-PCR، اكتشفنا عدم تكاثر الفيروس في كل من البعوضة المنزلية الشمالية *Culex pipiens pallens* وبعوضة الحمى الصفراء *Aedes aegyptis* وبعوضة *Anopheles sinensis*. كما فشلنا في عزل فيروسات بانيوية من خلايا الفيرو المزروعة بفيروسات بانيوية-البعوض المُصاب.

الخاتمة: تُظهر نتائج الدراسة الحالية إمكانية ضئيلة بأن يكون البعوض هو الناقل للفيروسات البانيوية للمرض.

Translated from English version into Arabic by Zeineb TRABELSI, through

TRANSLATORS WITHOUT BORDERS

蚊虫人工感染传播发热伴血小板减少综合征病毒的研究

梁姝怡,褚宏亮，郭喜玲，汪伟，陈红娜，张育富，陈银，吴涛，鲍倡俊，周明浩

摘要

引言：发热伴血小板减少综合征是一种由新布尼亚病毒引起的，最近在亚洲地区流行的新发传染病。虽然流行病学研究尚未提示蚊虫是发热伴血小板减少综合征的主要传播媒介，但是蚊虫在发热伴血小板减少综合征病毒人际传播中所扮演的角色并不明确。

发现：本研究中，我们通过蚊虫实验室人工感染发热伴血小板减少综合征病毒的方法，来探索蚊虫能否传播此新病毒。结果发现，通过 qRT-PCR 的方法，我们在淡色库蚊、白纹伊蚊和中华按蚊这三种蚊体中并没有检测到病毒增殖的情况。另外，病毒分离结果发现，感染的蚊虫研磨液接种细胞后未引起细胞病变。

结论：淡色库蚊、中华按蚊和埃及伊蚊不能作为发热伴血小板减少综合征病毒的传播媒介。

Translated from English version into Chinese by Shu-Yi Liang

Infections expérimentales de moustiques avec le virus du Syndrome de fièvre sévère avec thrombocytopénie

Shu-Yi Liang, Hong-Liang Chu, Xi-Ling Guo, Wei Wang, Hong-Na Chen, Yu-Fu Zhang, Yin Chen,

Tao Wu, Chang-Jun Bao, Ming-Hao Zhou

Résumé

Contexte: Le Syndrome de fièvre sévère avec thrombocytopénie (SFTS) est une maladie infectieuse émergente nouvellement identifiée, causée par un nouveau bunyavirus (nommée SFTSV), en Asie. Bien qu'il n'ait pas été établi que les moustiques soient les vecteurs principaux, comme il a été indiqué par des enquêtes épidémiologiques, leur rôle dans la transmission de ce SFTSV en tant que vecteur suspect n'a pas été validé.

Constatations: Dans le cadre de cette étude, nous avons réalisé des infections expérimentales de moustiques avec le SFTSV pour étudier leur rôle dans la transmission du virus. Nous n'avons pas décelé de réplication virale chez les espèces *Culex pipiens pallens, Aedes aegyptis et Anopheles sinensis,* comme ont démontré les résultats du test qRT-PCR. De plus, nous n'avons pas été en mesure d'isoler le SFTSV des cellules Vero cultivées avec des suspensions de moustiques infectés par le SFTSV.

Conclusion: Les résultats de cette étude démontrent qu'il est peu probable que les moustiques agissent en tant que vecteurs pour le pathogène émergent SFTSV.

Translated from English version into French by Edith Emilie Mercier, through

TRANSLATORS
WITHOUT BORDERS

Экспериментальное заражение комаров вирусом синдрома острой лихорадки с тромбоцитопенией

Шу-И Лян (Shu-Yi Liang), Хун-Лян Чу (Hong-Liang Chu), Си-Лин Го (Xi-Ling Guo), Вэй Ван (Wei Wang), Хун-На Чэнь (Hong-Na Chen), Юй-Фу Чжан (Yu-Fu Zhang), Инь Чэнь (Yin Chen), Тао У (Tao Wu), Чан-Цзюнь Бао (Chang-Jun Bao), Мин-Хао Чжоу (Ming-Hao Zhou)

Аннотация

Краткое описание. Синдром острой лихорадки с тромбоцитопенией (severe fever with thrombocytopenia syndrome, SFTS) – это недавно выявленная инфекционная болезнь в Азии, которая вызывается новым буньявирусом, получившим название SFTSV. Несмотря на то, что по результатам эпидемиологических обследований комары не были признаны первичными переносчиками инфекции, их роль в передаче вируса SFTSV в качестве возможного переносчика ещё не выяснена.

Результаты. В данном исследовании мы проводили экспериментальное заражение комаров вирусом SFTSV для выяснения их роли в передаче вируса. Мы не обнаружили репликации вируса у комаров *Culex pipiens pallens*, *Aedes aegyptis* и *Anopheles sinensis,* как показал метод qRT-PCR. Помимо этого, мы не смогли получить изолят SFTSV из клеток Vero с культурой суспензии, полученной от комаров, заражённых вирусом SFTSV.

Выводы. Результаты данного исследования продемонстрировали низкую вероятность того, что комары являются переносчиками недавно обнаруженного патогена SFTSV.

Translated from English version into Russian by Natalia Potashnik, through

TRANSLATORS WITHOUT BORDERS

Infecciones experimentales de mosquitos con el virus del síndrome de trombocitopenia con fiebre severa

Shu-Yi Liang, Hong-Liang Chu, Xi-Ling Guo, Wei Wang, Hong-Na Chen, Yu-Fu Zhang, Yin Chen, Tao Wu, Chang-Jun Bao, Ming-Hao Zhou

Resumen:
Antecedentes: El síndrome de trombocitopenia con fiebre severa (SFTS por sus siglas en inglés) es una nueva enfermedad infecciosa emergente en Asia, causada por un nuevo Bunyavirus (SFTSV por sus siglas en inglés). Aunque los mosquitos no han sido identificados como los vectores primarios, como revelan las encuestas epidemiológicas, no se ha confirmado que actúen como vectores en la transmisión del SFTSV.
Hallazgos: En este estudio, llevamos a cabo infecciones experimentales de mosquitos con SFTSV para examinar el papel de los mosquitos en la transmisión del virus. No hemos detectado la replicación viral en *Culex pipiens pallens, Aedes aegyptis y Anopheles sinensis* como ha revelado el ensayo qRT-PCR. Además, no se pudo aislar el SFTSV de las células Vero cultivadas con suspensiones de mosquitos infectados con SFTSV.
Conclusión: Los resultados del presente estudio revelan una posibilidad baja de que los mosquitos actúen como vectores para el patógeno emergente SFTSV.

Translated from English version into Spanish by Amparo Muñoz, through

TRANSLATORS WITHOUT BORDERS
